# Regulation of *Srpr* Expression by miR-330-5p Controls Proliferation of Mouse Epidermal Keratinocyte

**DOI:** 10.1371/journal.pone.0164896

**Published:** 2016-10-21

**Authors:** Bong-Kyu Kim, Hye-In Yoo, Keonwoo Choi, Ah-Reum Lee, Sungjoo Kim Yoon

**Affiliations:** Department of Medical Lifesciences, The Catholic University of Korea, Seoul, Korea; NYU Langone Medical Center, UNITED STATES

## Abstract

*Srpr* is a gene encoding α subunit of the signal recognition particle receptor which is involved in the targeting and translocation of nascent secretory and membrane proteins to the endoplasmic reticulum. Previous studies showed aberrant expression of *Srpr* in several cell types with abnormal growth rate. Although *Srpr* is expressed in various tissues including skin, the role of *Srpr* in keratinocytes and regulation of its expression by miRNAs have not been studied. In this study, we investigated the role of SRPR and regulation of its expression by miRNA in skin keratinocytes. We found that SRPR was highly expressed in epidermal keratinocytes and regulated keratinocyte proliferation by affecting cell cycle progression. We also demonstrated that miR-330-5p directly inhibits *Srpr* expression. These data suggest that miR-330-5p-mediated regulation of the SRPR level is needed for the regulation of proliferation of epidermal keratinocytes.

## Introduction

*Srpr* is a gene that encodes signal recognition particle receptor alpha (SRPRα, also called docking protein); it forms a heterodimer with SRPRβ, which becomes the signal recognition particle receptor (SR). SR is involved in the targeting and translocation of nascent secretory and membrane proteins to the endoplasmic reticulum membrane, which aids the spatial control of protein synthesis [1,2]. SR function is mediated in conjunction with the signal recognition particle (SRP) and is well conserved from bacteria to eukaryotes. SRP binds to the signal sequence of a newly synthesized peptide and slows protein synthesis (elongation arrest). This ribosome-nascent chain complex is targeted by SR to the protein-conducting channel, known as the translocon, in the endoplasmic reticulum membrane [3]. Aberrant *Srpr* expression has been detected in several cell types that have abnormal growth rate, suggesting that *Srpr* is essential for cell survival [4,5].

MicroRNAs (miRNAs) are non-coding RNA molecules (17–25 nucleotides) that regulate gene expression at the post-transcriptional level. This regulation is mediated through the recognition and annealing of complementary sequences (referred to as ‘seed’ regions) of the miRNAs to target sites, which comprise 6–8 nucleotides in the 3′ untranslated regions (UTRs) of the target mRNAs [6]. miRNAs play important roles in various biological processes including cell proliferation, apoptosis, and metastasis, and also affect sensitivity to chemotherapy and radiotherapy in multiple cancers [7,8]. In skin biology, the specific regulation of miRNAs is associated with keratinocyte proliferation, migration, and differentiation, and also with the development of skin disease. For example, up-regulation of miR-330-5p inhibits keratinocyte proliferation and migration by targeting *Pdia3* expression [9]. Overexpression of miR-199a-5p inhibits keratinocyte migration [10]. miR-378b promotes keratinocyte differentiation by targeting NKX3.1 [11]. Down-regulation of miR-31-5p accelerates hair follicle growth and alters hair shaft formation [12]. Therefore, it is considered that miRNAs have critical roles in skin biology and hair cycle regulation.

Although *Srpr* is expressed in various tissues including skin, the role of *Srpr* in keratinocytes and regulation of its expression by miRNAs have not been studied. In the present study, we investigated the role of *Srpr* in skin keratinocytes and miRNAs that regulate its expression in keratinocytes. We found that *Srpr* is abundantly expressed in epidermal keratinocytes and regulates their proliferation by promoting cell cycle progression. We also showed that miR-330-5p directly suppresses *Srpr* expression by targeting its 3′ UTR. These data suggest that miR-330-5p-mediated regulation of SRPR controls proliferation of epidermal keratinocytes.

## Materials and Methods

### Mice

The BALB/C mice (total 8 of male or female, P10-P49 of age, 15–25 g) were bred in the barrier system under specific-pathogen-free conditions with regulated light (07:00–19:00 h), temperature (23°C), humidity (50%), and ventilation (10–12 times per hour). In each cage, less than 5 mice were housed. Food and water were received *ad libitum*. To obtain the dorsal skin, each animal were sacrificed with CO2 inhalation. All animal experiments were approved by the Institutional Animal Care and Use Committee of the Catholic University of Korea. All experiments were carried out in accordance with the guidelines for animal experimentation and all efforts were made to minimize suffering.

### Cell culture and transfection experiment

PAM212 (mouse keratinocyte) and 3T3-L1 (mouse adipocyte) cells were maintained in Dulbecco's Modified Eagle Medium (Invitrogen) containing 10% fetal bovine serum with 5% CO_2_ in a 37°C incubator. For the inhibition of *Srpr* expression, total of 5×10^5^ cells were plated in a 60 mm dish. After 24 hrs, *Srpr* siRNA (Ambion) was transfected into these cells. MiR-330-5p mimic (Dharmacon) or miR-330-5p inhibitor (Dharmacon) was used for miR-330-5p over-expression or suppression, respectively. These experiments were carried out using DharmaFECT 1 transfection reagent (Dharmacon) following the manufacturer’s instruction. The negative mimic (Dharmacon) was used as control at the same concentration. At 72hr post-transfection, cells were harvested and used to extract total RNA or protein. For the over-expression of *Srpr*, the full length *Srpr*-cDNA construct was purchased (Sino biological) and transfected into cells using the Lipofectamine 2000 reagent. All experiments were repeated in triplicate. Cell images were captured at three different fields which were located at the relatively similar position in each plate using inverted microscope (Leica).

### Cell proliferation assay

PAM212 cells (2×10^4^) were seeded in a 96-well plate and incubated for 24 hrs at 37°C. Transfection experiments were performed with *Srpr* siRNA or the miR-330-5p mimic using DharmaFECT 1 transfection reagent. After 72 hrs, the relative viability of the cells was measured using an EZ-Cytox Cell viability assay kit which measures mitochondrial reduction of WST-8 (2-(2-methoxy-4-nitrophenyl)-3-(4-nitrophenyl)-5-(2,4-disulfophenyl)-2H-tetrazolium, monosodium salt) (DoGen) according to the manufacturer’s protocol.

### RT-PCR and Real-time PCR

Total RNAs were prepared from the cells or dorsal skins of BALB/C mice of various ages using the QIAzol reagent (Qiagen). Then cDNA was synthesized using the PrimeScript 1st strand cDNA Synthesis kit (Takara). Realtime PCR was carried out with the SYBR Premix Ex Taq (Takara) in an Mx3000P (Stratagene) and the annealing temperatures given in the Table 1. All the expression levels were normalized against glyceraldehyde-3-phosphatedehydrogenase gene (*Gapdh*) expression using the comparative ΔΔCt method [13]. Results are the average of three independent experiments performed in duplicate.

**Table 1:** List of gene specific primers for Realtime PCR.

Genes	Accession Number	Sequences	size (bp)	Tm (°C)
*Srpr*	NM_026130.1	F: atgatgaaggggccactcaa	171	60
		R cagcagccacattcttagca		
*Srpr *(3’UTR)**	NM_026130.1	F: atgtggctcttgcctaatacca	908	62
		R: atttctctgggcagacagcc		
*SRPR*	NM_003139.3	F: cagaagcatgggaggggtat	200	60
		R: ccagtccctcgaatcaggtt		
*Gapdh*	NM_008084	F: aactttggcattgtggaagg	223	62
		R: acacattgggggtaggaaca		
*GAPDH*	NM_002046	F: gagtcaacggatttggtcgtt	238	60
		R: ttgattttggagggatctcg		

### Western blot analysis

Total lysated PAM212 cells were prepared using radioimmunoprecipitation assay buffer (150 mM sodium chloride, 1% NP-40, 0.5% sodium deoxycholate, 0.1% sodium dodecyl sulfate, 50 mM Tris-HCl [pH 8.0]) following the standard method at 72 hrs post-transfection. Total eighty micrograms of protein were loaded to 10% sodium dodecyl sulfate polyacrylamide gel electrophoresis and transferred to a nitrocellulose membrane. This membrane was incubated with a rabbit polyclonal SRPR antibody (1:1000, Abcam) or a mouse polyclonal GAPDH antibody (1:5000, Applied Biological Materials) following the standard protocol. The signal was detected using an enhanced chemiluminescence system (Amersham Bioscience). The relative quantities of proteins were determined using ImageJ software (http://imagej.nih.gov/ij/index.html).

### Plasmid Construction

The cDNAs of the full length 3’ UTR of *Srpr* was amplified from the dorsal skin cDNA of wild-type mice by PCR using PrimeSTAR DNA Polymerase (Takara). The gene-specific primers are listed in the Table 1. The product was cloned into pGEMT-easy vector and subsequently subcloned into the psiCHECK-2 vector (Promega) using *Not* I site.

### Dual luciferase reporter assay

For luciferase assay, PAM212 cells (5 × 10^5^/dish) were plated in 60 mm dishes and incubated for 24 hrs at 37°C. Then the construct containing the full length 3’ UTR of *Srpr* (+1987 bp -+2904 bp, Table 1) was transfected into cells with miR-330-5p mimic or control mimic using the Lipofectamine 2000 reagent. Luciferase activity was measured at 48 hrs post-transfection using the Dual-Luciferase Reporter Assay reagent (Promega).

### Cell cycle assay

*Srpr* siRNA treated PAM212 cells were collected at 72hr post-transfection and washed with PBS twice. Then cells were fixed in 70% ethanol at 4°C overnight and incubated in 1 mg/ml RNase A at 37°C for 30min. These cells were re-suspended in a propidium iodide staining solution (50 μg/ml). The distribution of cells in each phase of the cell cycle was measured using FACSCanto II (BD Biosciences).

### Immunohistochemistry

Mouse dorsal skins of BALB/C mice at postnatal days 7 (P7), 17 (P17), 21 (P21), 28 (P28), 35 (P35), 42 (P42) and 49 (P49) were prepared and each tissue section was deparaffinized, rehydrated, and washed with PBS, sequentially. Then, antigen retrieval was carried out by treating slides with 10 mM sodium citrate buffer for 15 min. Subsequently, the slides were incubated with an antibody against SRPR (1:100, Thermofisher) for overnight at room temperature. After washing three times with PBS, each slide was incubated with a horseradish peroxidase-conjugated antibody (Dako) for 1 hr at room temperature. The signals were detected with a fluorescent microscope (Olympus) after a color reaction using diaminobenzidine as a chromogen (Dako).

### Statistical analysis

Statistical significance was determined by Student's *t*-tests and values < 0.05 were regarded as statistically significant.

## Results

### SRPR was expressed in epidermal keratinocyte in mouse skin

**Fig 1:**
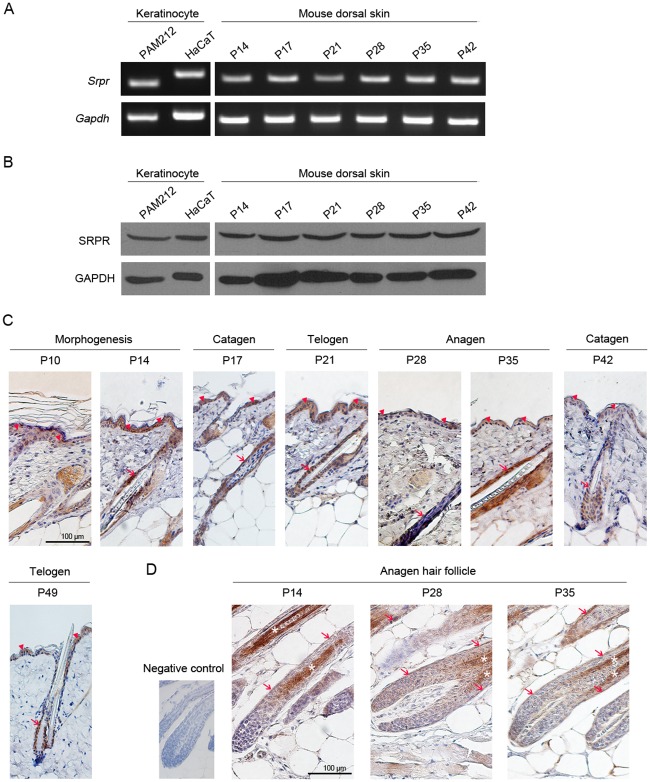
Abundant expression of SRPR in mouse epidermal keratinocyte. (A-B) *Srpr* was abundantly expressed in keratinocyte (PAM212 and HaCaT cell) and mouse dorsal skin at both mRNA by RT-PCR (A) and protein level by western blot analysis (B). (C-D) SRPR expression in dorsal skin (C) and HF (D) of BALB/C mice at postnatal days P10, P14, P17, P21, P28, P35, P42 and P49 by immunohistochemistry. Brown signals indicated the SRPR-positive epidermis cells (arrowhead), HF (arrow) and hair cortex (star). Scale bar = 100 μm.

To determine whether SRPR has a function in keratinocytes, we first examined the *Srpr* expression in keratinocytes and mouse dorsal skin. We used mouse (PAM212) and human (HaCaT) keratinocyte cell lines and mouse dorsal skin at various hair cycle stages (P10, P14, P17, P21, P28, P35, and P42). RT-PCR and western blot analysis revealed that both *Srpr* mRNA and the SRPR protein were abundantly expressed in both keratinocytes and mouse dorsal skin (Fig 1A and 1B). In addition, we performed real-time PCR to compare expression level of *Srpr* with those of several genes whose expressions were regulated by miR-330-5p in keratinocyte [9]. As shown in the S1 Fig, *Srpr* was abundantly expressed to the level similar to that of *Integrin5A*. Next, we performed immunohistochemistry to further analyze the expression pattern of SRPR in wild-type mouse dorsal skin and found the strong SRPR expression in the interfollicular epidermis, hair cortex, and outer root sheath (ORS) at all hair cycle stages (Fig 1C and 1D). In contrast, we did not find SRPR expression in the hair matrix, or dermis including dermal papillae. These results indicate that SRPR is mainly expressed in epidermal keratinocytes in mouse skin.

### *Srpr* knockdown induced inhibition of proliferation of mouse keratinocyte

**Fig 2:**
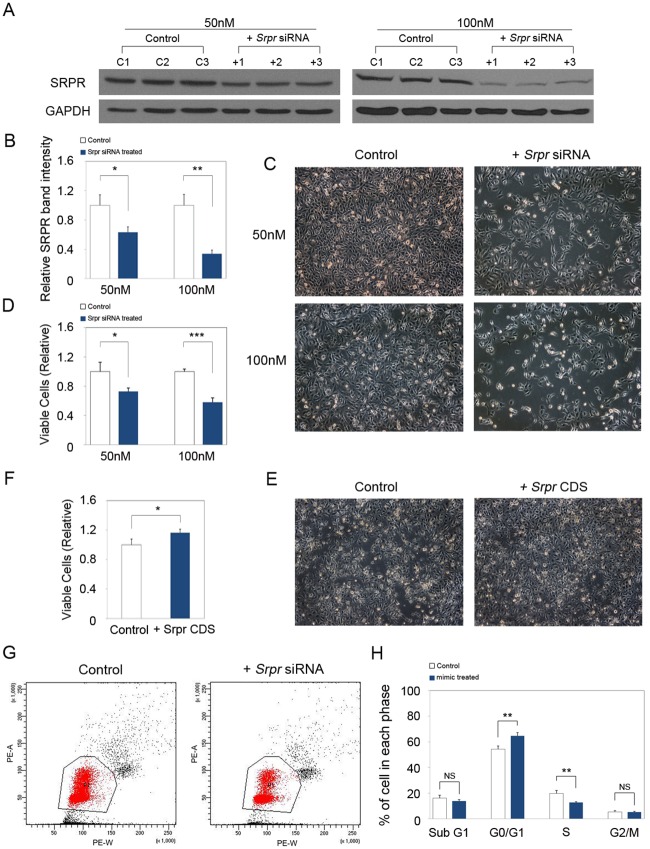
SRPR regulates the proliferation of keratinocyte. (A) Western blot analysis showed that SRPR protein expression was significantly decreased at 50nM and 100nM *Srpr* siRNA transfected PAM212 cells. GAPDH was used as a loading control. (B) Quantitative analysis of western blot using ImageJ software. The data was normalized against GAPDH expression. (C) Proliferation assay revealed that *Srpr* siRNA transfection inhibited proliferation of PAM212 cells in comparison with control at both 50 nM and 100 nM in a dose-dependent manner. (D) Relative viable cells were measured at 48 h after transfection. (E, F) In contrast, relative viable PAM212 cells were increased by transfection of Srpr CDS construct. (G, H) Cell cycle assay was performed and revealed that *Srpr* knockdown induced cell cycle arrest at G0/G1 and number of cell at S phase were decreased. (A-H) Results are the average of three independent experiments *P<0.05; **P<0.01; ***P<0.001. NS = no significant.

SRPR was previously shown to be important for cell growth in *Saccharomyces cerevisiae* [4]. As cells in the basal layer of the epidermis and in the ORS continuously proliferate throughout the lifetime, we hypothesized that SRPR affects keratinocyte proliferation in the mouse skin. To test this hypothesis, we utilized a mouse keratinocyte cell line, PAM212 as an experimental system. We transfected PAM212 cells with *Srpr* siRNA (50 or 100 nM). Western blot analysis confirmed a decreased expression of SRPR in *Srpr* siRNA-transfected cells in comparison with the control cells at both siRNA concentrations (Fig 2A and 2B). Proliferation assays showed that SRPR knockdown inhibited the proliferation of PAM212 cells transfected with either 50 nM or 100 nM siRNA in a concentration-dependent manner (Fig 2C and 2D). To further investigate whether keratinocyte proliferation is affected by the SRPR expression level, we induced SRPR expression by transfecting PAM212 cells with a *Srpr* cDNA expression construct. The total number of PAM212 cells increased in comparison with that in the mock transfection control (Fig 2E and 2F).

Next, we examined the cell cycle distribution of the cells to determine whether inhibition of proliferation by decreased SRPR expression was caused by cell cycle arrest. The number of cells in G0/G1 phase was significantly higher and that in S phase was markedly lower among cells transfected with *Srpr* siRNA than among the control cells (Fig 2G and 2H). This data suggests that SRPR plays a role in cell proliferation by promoting the progression from G1 to S phase of the cell cycle.

### MiR-330-5p regulates endogenous *Srpr* expression in mouse keratinocyte

**Fig 3:**
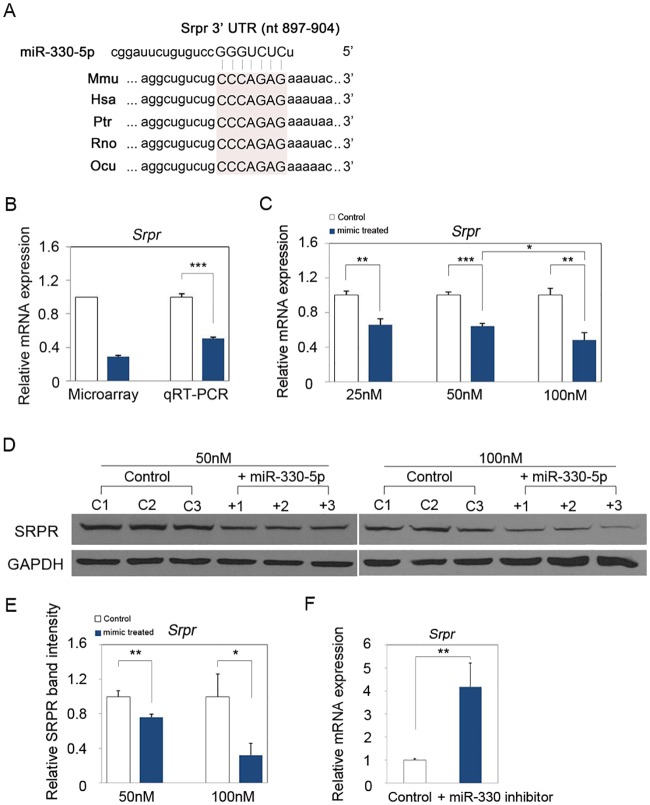
MiR-330-5p regulates *Srpr* expression in mouse epidermal keratinocyte. (A) Targetscan algorithm predicted that a conserved binding sequence of miR-330-5p was present in the 3’ UTR of *Srpr* mRNA. (B) Real-time quantitative PCR was performed to validate the result of previous study using the same total RNA used for microarray analysis. (C) MiR-330-5p down-regulated the *Srpr* expression at 25nM, 50nM and 100nM mimic transfected PAM212 cells. The data was normalized against *Gapdh* expression. (D) Western blot analysis also revealed that SRPR protein level was decreased at 50nM and 100nM miR-330-5p mimic transfected PAM212 cells in comparison to the control. β-actin was used as a loading control. (E) Quantitative analysis of western blot using ImageJ software. The SRPR band intensity was normalized against GAPDH expression. (F) *Srpr* expression was significantly increased by inhibition of miR-330-5p. (B-F) Results are the average of three independent experiments. *P<0.05; **P<0.01; ***P<0.001.

Using microarray analysis, we recently reported that expression of many genes was significantly changed in miR-330-5p-overexpressing PAM212 cells compared to the control [9]. In particular, we found that *Srpr* expression was down-regulated by miR-330-5p. In line with this finding, the 3′ UTR of *Srpr* mRNA contains a seed sequence for miR-330-5p, which is conserved in various species (Fig 3A). We previously showed that miR-330-5p inhibits proliferation of mouse keratinocytes [9] and have confirmed this effect in the present study (S2 Fig). On the basis of these observations, we hypothesized that the regulation of cell proliferation by SRPR might be mediated by miR-330-5p. To test this hypothesis, we first validated *Srpr* down-regulation by miR-330-5p using real-time quantitative PCR on total RNAs that were used for microarray analysis in our previous study [9]. We found that the expression of *Srpr* mRNA was significantly decreased in PAM212 cells transfected with a miR- 330-5p mimic than in the control-transfected cells (Fig 3B). Next, we analyzed the effect of miR-330-5p on *Srpr* expression in keratinocytes at the mRNA and protein levels using real-time quantitative PCR and western blotting, respectively. Expression of *Srpr* mRNA was consistently decreased in PAM212 cells transfected with the miR-330-5p mimic in comparison with control-transfected cells (Fig 3C). Similarly, SRPR expression was reduced by miR-330-5p mimic treatment (Fig 3D and 3E). In order to confirm these findings, we also investigated the expression of *Srpr* using a miR-330-5p inhibitor. We found that transfection of miR-330-5p inhibitor led to a significant induction of the endogenous *Srpr* expression (Fig 3F). These data indicate that SRPR expression is regulated by miR-330-5p at both mRNA and protein levels.

### 3’ UTR of *Srpr* is a direct target of miR-330-5p in mouse keratinocyte

**Fig 4:**
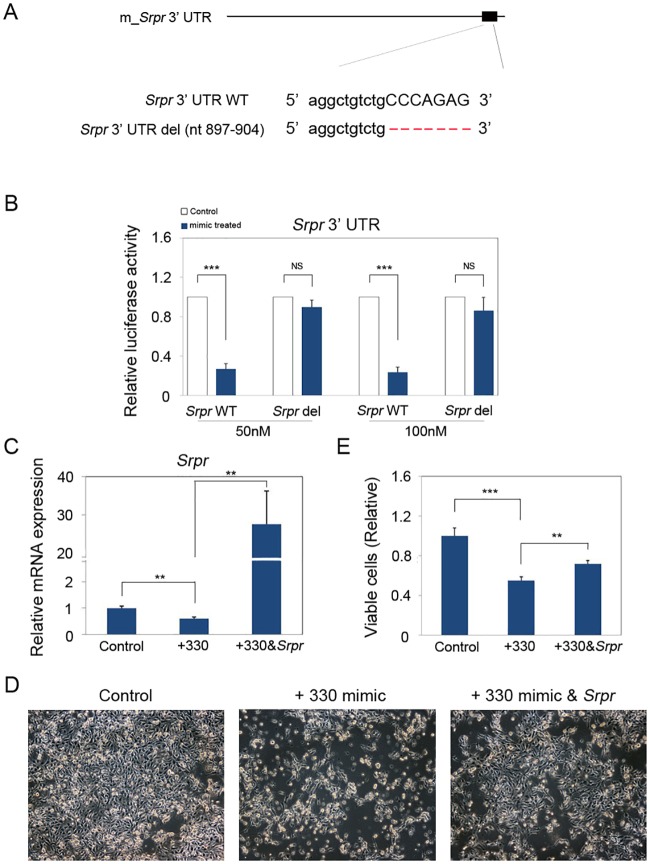
*Srpr* is a direct target of miR-330-5p in mouse keratinocyte. (A) Location of the binding site for miR-330-5p in 3’UTR of *Srpr* mRNA. Deletion is depicted in red hyphen. (B) Dual Luciferase assay showed that miR-330-5p directly inhibited the luciferase activity by targeting the binding site in *Srpr* 3’UTR. However, luciferase activity did not change significantly when cells were co-transfected with the deletion mutant lacking the miR-330-5p-binding site and the miR-330-5p. (C-E) Mir-330-5p suppressed the proliferation of PAM212 cells through inhibition of SRPR expression. (C) Real-time quantitative PCR showed that cotransfection of miR-330-5p and the full length SRPR-cDNA (*Srpr* CDS) was able to abolish the decreased expression of Srpr by miR-330-5p alone. (D) Proliferation of PAM212 cells was decreased by miR-330-5p. Co-transfection with the *Srpr* CDS construct rescued inhibition of cell proliferation by miR-330-5p. (E) Relative viable cells were measured at 72 h after transfection. (B-E) Results are the average of three independent experiments. **P<0.01; ***P<0.001. NS = no significance.

The web-based target prediction software programs (TargetScan and microRNA.org) suggested that a binding site for miR-330-5p is present in the 897–904 bp region of the *Srpr* 3′ UTR (Fig 4A). To examine whether SRPR expression is directly regulated by miR-330-5p, we performed a luciferase reporter assay using constructs generated in the psiCHECK-2 dual luciferase vector. As shown in Fig 4B, the wild-type construct with full-length 3′ UTR showed significantly decreased luciferase activity when cells were co-transfected with the miR-330-5p mimic at both 50 nM and 100 nM concentrations. However, luciferase activity did not change significantly when cells were co-transfected with the deletion mutant lacking the miR-330-5p binding site and the miR-330-5p (Fig 4B). These data suggest that *Srpr* is a direct target of miR-330-5p.

Finally, we used proliferation assay to investigate whether miR-330-5p inhibits keratinocyte proliferation by targeting SRPR expression. We compared proliferation of cells transfected with the miR-330-5p mimic alone or co-transfected with the full length SRPR-cDNA (*Srpr* CDS) construct and the miR-330-5p mimic. Co-transfection with the *Srpr* CDS construct rescued inhibition of cell proliferation by miR-330-5p (Fig 4C–4E). Therefore, we conclude that SRPR mediates the inhibitory effect of miR-330-5p on mouse keratinocyte proliferation.

## Discussion

For sustained cell growth, cellular machinery for targeting nascent secretory proteins and membrane proteins to their proper ER locations is essential. SRP and SRPR are part of this cellular machinery and play a critical role in this process [14]. In the present study, we found that SRPR is abundantly expressed in epidermal keratinocytes, including those in the interfollicular epidermis and ORS. The epidermis is a continuously regenerating tissue derived from transient amplifying progenitors located in its basal layer. ORS is similar to the epidermis in that it is formed by proliferation of progenitor cells located in the bulge; ORS plays an indirect paracrine role in the regulation of hair growth [15,16]. These notions suggest that SRPR is expressed in proliferating keratinocytes and may modulate their proliferation. In addition, SRPR expression does not seem to be limited to basal cells. SRPR expression is also present in the suprabasal layer cells (Fig 1C). Thus, SRPR may play another role in epidermis such as differentiation, cell-cell communication and so on.

Together with SRP, SRPR as a docking protein involves in recognition and translocation of proteins to ER membrane. Thus SRPR plays a basic function for the cell. In the current study, we found that *Srpr* knockdown inhibited proliferation of mouse keratinocytes in vitro by arresting cell cycle at G0/G1 phase, thus reducing the number of cells in S phase. Moreover, this cell cycle arrest did not increased the apoptotic cell death (Fig 2H). Although G0/G1 arrest is a common response in cells that are perturbed by a variety of means, our results suggest that SRPR directly or indirectly controls cell proliferation by promoting the progression of cells from G1 to S phase. Our finding is in line with the previous report on the relationship between SRPR and retinoblastoma protein (pRb), an important regulator of cell proliferation and differentiation. In G0/G1 phase, pRb was shown to bind the *Srpr* promoter together with E2F1, an S phase-promoting transcription factor; binding of these proteins showed inhibition of *Srpr* transcription in human leukemia cells [17]. pRb restricts DNA replication by preventing progression from G1 to S phase [18]. Thus, collectively these findings suggest that SRPR plays a role in the regulation of cell proliferation by affecting the cell cycle, specifically transition from G1 to S phase. Further mechanistic studies will be needed in order to elucidate the mechanisms how SRPR affects these processes in keratinocytes.

A similar observation has been reported in *S*. *cerevisiae*, in which *Srpr* disruption decreased growth rate [4], suggesting that SRPR is required for cell proliferation. On the contrary, down-regulation of *Srpr* was detected in a rat model of cyclosporine A-induced gingival overgrowth [5], suggesting that SRPR may not be the only one ER targeting regulator affecting cell proliferation. While there are no other loci known for *Srpr* in mouse, rat and human, there are 2 isoforms reported in both rat and human. Thus, it raises a possibility that an isoform may play a redundant function in rat and human. Obviously, a more detailed examination of the function of SRPR in cell proliferation is needed to elucidate this complex mechanism.

Previous reports indicated that deregulated miR-330-5p expression controls cell proliferation in colorectal and prostate cancers [19,20]. Recently, we also showed that miR-330-5p inhibits keratinocyte proliferation by arresting cells in G0/G1 phase [9]. Moreover, in this study, we demonstrated that miR-330-5p directly down-regulates the expression of SRPR by targeting its 3′ UTR, suggesting that the inhibitory effect of SRPR down-regulation on cell proliferation is mediated by miR-330-5p. Similar to SRPR, knockdown of *Pdia3* expression by miR-330-5p inhibits proliferation of mouse keratinocytes [9], which explains why *Srpr* overexpression did not fully rescue the inhibitory effect of miR-330-5p on keratinocyte proliferation. Therefore, miR-330-5p is a key regulator of cell cycle because it targets genes associated with this process.

We also showed that decreased expression of *Srpr* inhibits proliferation of 3T3-L1 adipocytes (S3 Fig). In this cell line, *Srpr* expression was also down-regulated by miR-330-5p through direct binding to its 3′ UTR (S4 Fig). These results raise a possibility that regulation of *Srpr* by miR-330-5p occurs in various cell types and plays important roles in regulation of cell proliferation. Further studies are required to elucidate the detailed mechanisms underlying these regulatory effects.

In summary, our current study suggests that SRPR plays a critical role in keratinocyte proliferation by affecting the cell cycle. This effect of SRPR is controlled by miR-330-5p. These findings provide evidence for a new role of SRPR and miR-330-5p in keratinocytes and, more generally, in skin biology.

## Supporting Information

S1 Fig Comparison of *Srpr* expression with several genes that regulated by miR-330-5p in keratinocyte. Real-time PCR was performed to compare expression level of *Srpr* with several genes whose expressions were regulated by miR-330-5p in keratinocyte. Results are the average of three independent experiments.
(DOC)

S2 Fig Inhibition of proliferation by miR-330-5p. (A) Over-expression of miR-330-5p inhibited proliferation of PAM212 cells in a dose dependant manner. (B) Relative cell viability was determined at 72 h post transfection with 50 nM and 100 nM RNAs. Results are the average of three independent experiments. *P<0.05; ***P<0.001.
(DOC)

S3 Fig Decreased expression of *Srpr* also inhibited proliferation of 3T3-L1 cells. (A) *Srpr* siRNA transfection induced the inhibition of proliferation. (B) Relative viable cells were measured after 48 h transfection. Results are the average of three independent experiments. **P<0.01; ***P<0.001.
(DOC)

S4 Fig *Srpr* is a target of miR-330 in mouse 3T3-L1 cells. (A) MiR-330-5p over-expression induced Proliferation inhibition of the 3T3-L1 cells. (B) Relative viable cells were counted after 72 h transfection. (C) MiR-330-5p down-regulated the *Srpr* expression in 3T3-L1 cells. The data was normalized against *Gapdh* expression. (D) Dual Luciferase assay revealed that miR-330-5p significantly inhibited the luciferase activity in the 3T3-L1 cells containing full length of *Srpr* 3’UTR. (B-D) Results are the average of three independent experiments. **P<0.01; ***P<0.001.
(DOC)
