# Supplementary material for: Regulation of Srpr Expression by miR-330-5p Controls Proliferation of Mouse Epidermal Keratinocyte
Source: PLoS One. 2016 Oct 21;11(10):e0164896. doi: 10.1371/journal.pone.0164896 (PMC5074476; doi:10.1371/journal.pone.0164896)
Supplement: S1 Fig — Real-time PCR was performed to compare expression level of Srpr with several genes whose expressions were regulated by miR-330-5p in keratinocyte. Results are the average of three independent experiments. (DOC) [file pone.0164896.s001.doc]

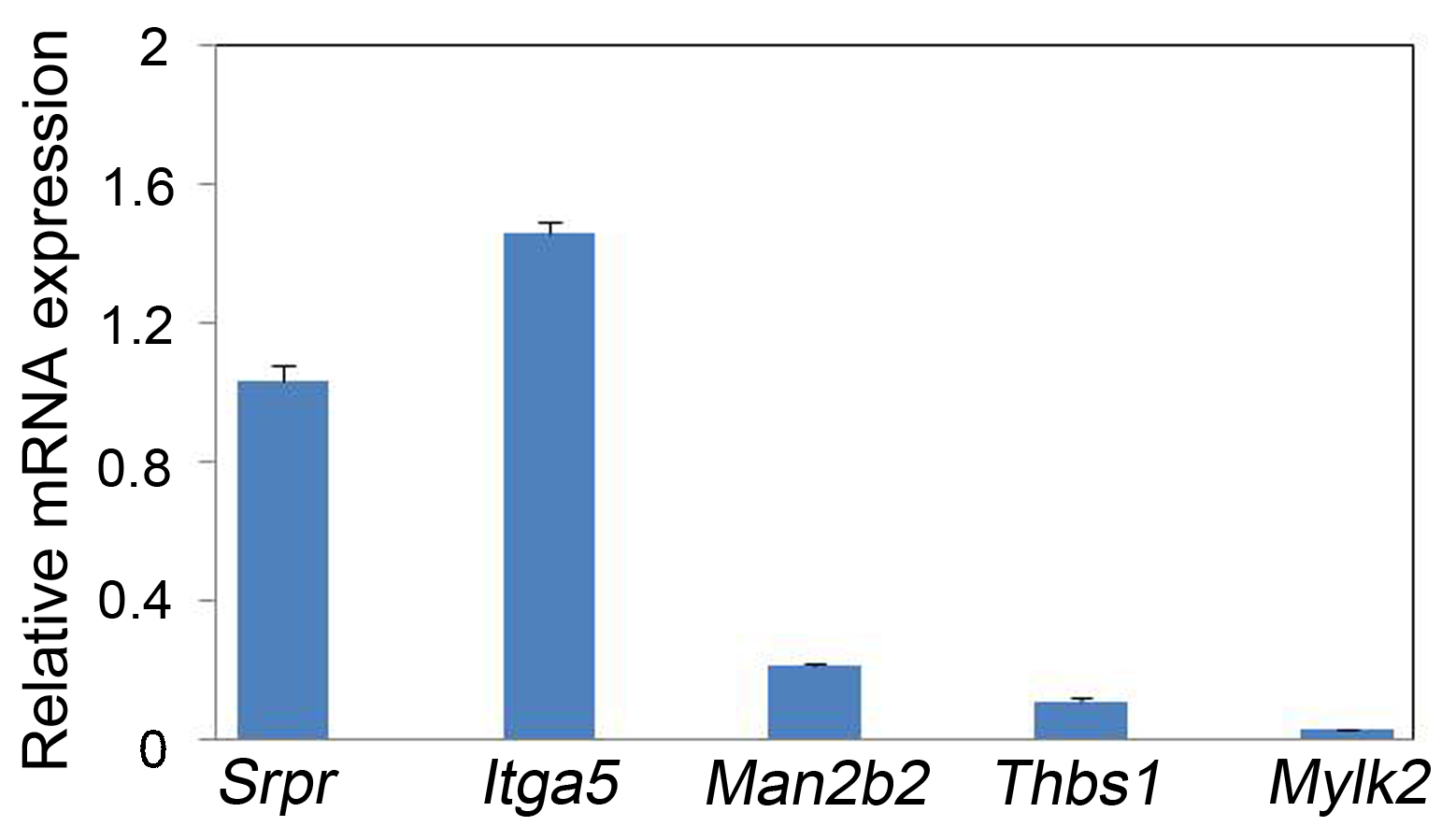


**S1 Fig. Comparison of *Srpr* expression with several genes that regulated by miR-330-5p in keratinocyte.** Real-time PCR was performed to compare expression level of *Srpr* with several genes whose expressions were regulated by miR-330-5p in keratinocyte. Results are the average of three independent experiments.
