# Supplementary material for: Regulation of Srpr Expression by miR-330-5p Controls Proliferation of Mouse Epidermal Keratinocyte
Source: PLoS One. 2016 Oct 21;11(10):e0164896. doi: 10.1371/journal.pone.0164896 (PMC5074476; doi:10.1371/journal.pone.0164896)
Supplement: S2 Fig — (A) Over-expression of miR-330-5p inhibited proliferation of PAM212 cells in a dose dependant manner. (B) Relative cell viability was determined at 72 h post transfection with 50 nM and 100 nM RNAs. Results are the average of three independent experiments. *P<0.05; ***P<0.001. (DOC) [file pone.0164896.s002.doc]

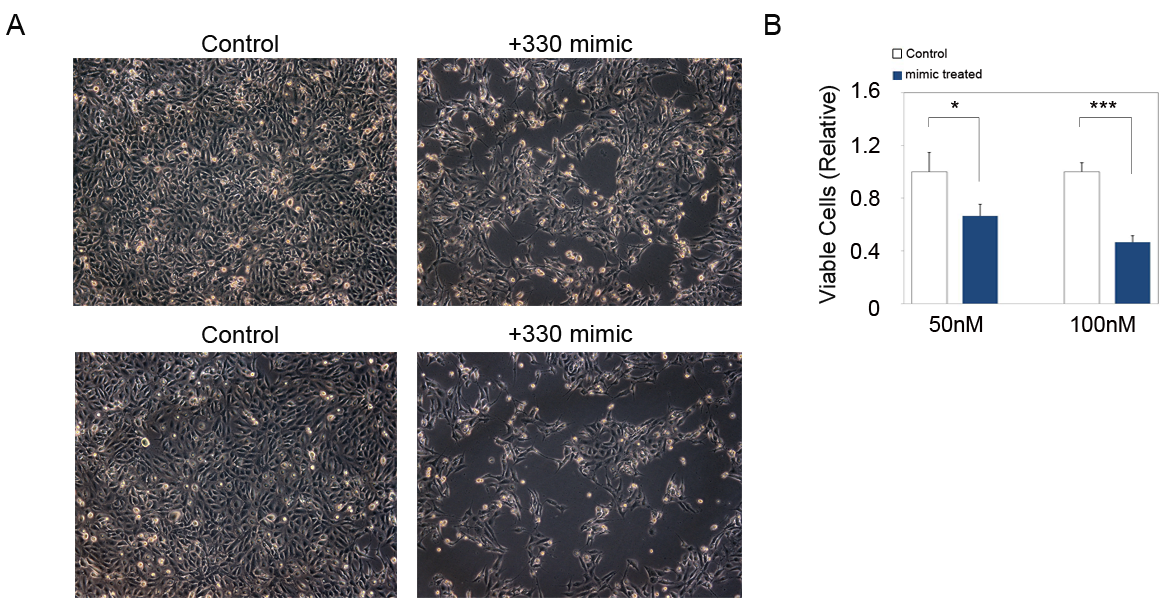


**S2 Fig. Inhibition of proliferation by miR-330-5p.** (A) Over-expression of miR-330-5p inhibited proliferation of PAM212 cells in a dose dependant manner. (B) Relative cell viability was determined at 72 h post transfection with 50 nM and 100 nM RNAs. Results are the average of three independent experiments. *P<0.05; ***P<0.001.
