# Supplementary material for: Regulation of Srpr Expression by miR-330-5p Controls Proliferation of Mouse Epidermal Keratinocyte
Source: PLoS One. 2016 Oct 21;11(10):e0164896. doi: 10.1371/journal.pone.0164896 (PMC5074476; doi:10.1371/journal.pone.0164896)
Supplement: S3 Fig — (A) Srpr siRNA transfection induced the inhibition of proliferation. (B) Relative viable cells were measured after 48 h transfection. Results are the average of three independent experiments. **P<0.01; ***P<0.001. (DOC) [file pone.0164896.s003.doc]

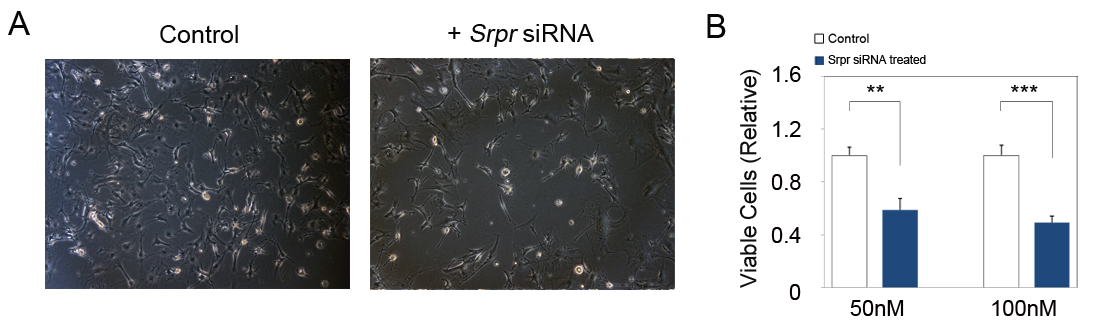


**S3 Fig. Decreased expression of *Srpr* also inhibited proliferation of 3T3-L1 cells.** (A) *Srpr* siRNA transfection induced the inhibition of proliferation. (B) Relative viable cells were counted after 48 h transfection. Results are the average of three independent experiments. **P<0.01; ***P<0.001.
