# Supplementary material for: Regulation of Srpr Expression by miR-330-5p Controls Proliferation of Mouse Epidermal Keratinocyte
Source: PLoS One. 2016 Oct 21;11(10):e0164896. doi: 10.1371/journal.pone.0164896 (PMC5074476; doi:10.1371/journal.pone.0164896)
Supplement: S4 Fig — (A) MiR-330-5p over-expression induced Proliferation inhibition of the 3T3-L1 cells. (B) Relative viable cells were counted after 72 h transfection. (C) MiR-330-5p down-regulated the Srpr expression in 3T3-L1 cells. The data was normalized against Gapdh expression. (D) Dual Luciferase assay revealed that miR-330-5p significantly inhibited the luciferase activity in the 3T3-L1 cells containing full length of Srpr 3’UTR. (B-D) Results are the average of three independent experiments. **P<0.01; ***P<0.001. (DOC) [file pone.0164896.s004.doc]

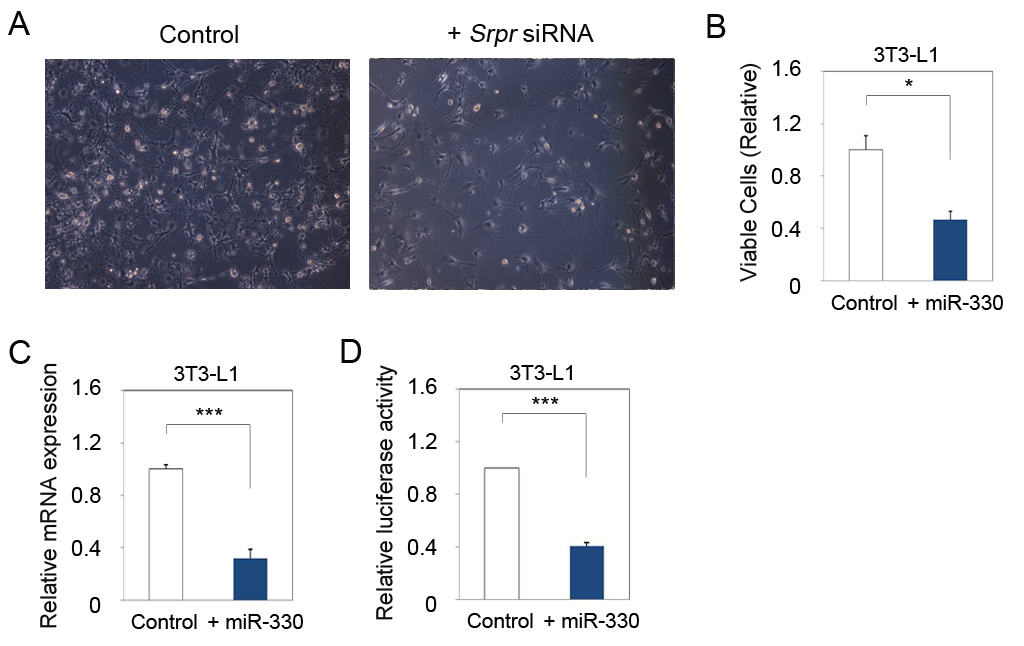


**S4 Fig. *Srpr* is a target of miR-330 in mouse 3T3-L1 cells.** (A) MiR-330-5p over-expression induced Proliferation inhibition of the 3T3-L1 cells. (B) Relative viable cells were counted after 72 h transfection. (C) MiR-330-5p down-regulated the *Srpr* expression in 3T3-L1 cells. The data was normalized against *Gapdh* expression. (D) Dual Luciferase assay revealed that miR-330-5p significantly inhibited the luciferase activity in the 3T3-L1 cells containing full length of *Srpr* 3’UTR. (B-D) Results are the average of three independent experiments. **P<0.01; ***P<0.001.
